# Subphenotypes in patients with acute respiratory distress syndrome treated with high-flow oxygen

**DOI:** 10.1186/s13054-023-04687-0

**Published:** 2023-11-01

**Authors:** Pierre-Louis Blot, Benjamin G. Chousterman, Manel Santafè, Jérôme Cartailler, Andrés Pacheco, Mònica Magret, Joan R. Masclans, Antoni Artigas, Oriol Roca, Marina García-de-Acilu

**Affiliations:** 1 Département d’anesthésie-Réanimation, Hôpital Lariboisière, Paris, France; 2 INSERM UMRS-942 MASCOT, Hôpital Lariboisière, Paris, France; 3 Servei de Medicina Intensiva, Parc Taulí Hospital Universitari, Institut de Recerca Part Taulí (I3PT-CERCA), Parc del Taulí 1, 08028 Sabadell, Spain; 4 Servei de Medicina Intensiva, Hospital Universitari Vall d’Hebron, Barcelona, Spain; 5 Servei de Medicina Intensiva, Hospital Universitari Joan XXIII, Tarragona, Spain; 6 Critical Care Department, Hospital del Mar-Parc de Salut MAR. GREPAC-Group Recerca Departamento de Medicina y Ciencias de la Vida Universitat Pompeu Fabra (UPF), Barcelona, Spain; 7 Director de Docencia PSMAR, Intensive Care Unit Hospital del Mar. Professor of Medicine Universitat Pompeu Fabra (UPF) IMIM (GREPAC - Group Recerca Patologia Critica) Departamento de Medicina Y Ciencias de la Vida (MELIS), Universitat Pompeu Fabra, Barcelona, Spain; 8 Departament de Medicina, Universitat Autònoma de Barcelona, Bellaterra, Spain; 9 CIBER de Enfermedades Respiratorias, Insituto de Salud Carlos III, Madrid, Spain

**Keywords:** High-flow nasal cannula, High-flow nasal oxygen, Acute respiratory distress syndrome, Inflammation, Subphenotypes, Biomarkers, Non-invasive ventilation

## Abstract

**Background:**

Acute respiratory distress syndrome (ARDS) subphenotypes differ in outcomes and treatment responses. Subphenotypes in high-flow nasal oxygen (HFNO)-treated ARDS patients have not been investigated.

**Objectives:**

To identify biological subphenotypes in HFNO-treated ARDS patients.

**Methods:**

Secondary analysis of a prospective multicenter observational study including ARDS patients supported with HFNO. Plasma inflammation markers (interleukin [IL]-6, IL-8, and IL-33 and soluble suppression of tumorigenicity-2 [sST2]) and lung epithelial (receptor for advanced glycation end products [RAGE] and surfactant protein D [SP-D]) and endothelial (angiopoietin-2 [Ang-2]) injury were measured. These biomarkers and bicarbonate were used in K-means cluster analysis to identify subphenotypes. Logistic regression was performed on biomarker combinations to predict clustering. We chose the model with the best AUROC and the lowest number of variables. This model was used to describe the HAIS (High-flow ARDS Inflammatory Subphenotype) score.

**Results:**

Among 41 HFNO patients, two subphenotypes were identified. Hyperinflammatory subphenotype (*n* = 17) showed higher biomarker levels than hypoinflammatory (*n* = 24). Despite similar baseline characteristics, the hyperinflammatory subphenotype had higher 60-day mortality (47 vs 8.3% *p* = 0.014) and longer ICU length of stay (22.0 days [18.0–30.0] vs 39.5 [25.5–60.0], *p* = 0.034). The HAIS score, based on IL-8 and sST2, accurately distinguished subphenotypes (AUROC 0.96 [95%CI: 0.90–1.00]). A HAIS score ≥ 7.45 was predictor of hyperinflammatory subphenotype.

**Conclusion:**

ARDS patients treated with HFNO exhibit two biological subphenotypes that have similar clinical characteristics, but hyperinflammatory patients have worse outcomes. The HAIS score may identify patients with hyperinflammatory subphenotype and might be used for enrichment strategies in future clinical trials.

**Supplementary Information:**

The online version contains supplementary material available at 10.1186/s13054-023-04687-0.

## Background

Acute respiratory distress syndrome (ARDS) is a common condition in ICU, carrying a high mortality burden. Recent research and the latest guidelines on ARDS management underlined the importance of identifying clinical and biological features to classify ARDS patients into subphenotypes that might have different outcomes and respond differently to specific therapies [1]. Identification of subphenotypes in ARDS mechanically ventilated patients has mainly relied on inflammation-related biomarkers. Consistently across studies, two subphenotypes have been reported, termed hypoinflammatory and hyperinflammatory, that have different outcomes and distinct responses to clinical interventions [2–7]. The stability of the subphenotypes over time was also shown in the same individual [8]. More recently, these subphenotypes were also identified in a large cohort of patients with COVID-19-associated ARDS [9]. Differentiating hypoinflammatory and hyperinflammatory ARDS enables prognosis enrichment and enhances the likelihood of treatment response [10].

Since the use of high-flow nasal oxygen (HFNO) in adult patients with hypoxemic acute respiratory failure was first described [11], its use has continuously increased [12–14]. Traditionally, patients treated with HFNO with bilateral opacities in the chest X-ray and a PaO_2_/FiO_2_ ratio ≤ 300 mmHg were not considered ARDS patients as they were not on positive pressure ventilation. However, some studies including non-intubated patients who meet all other ARDS criteria showed that they had similar biological characteristics [15] and clinical outcomes [16] to mechanically ventilated ARDS patients. Similarly, the results of a recent study in COVID-19 patients showed that more than 90% of the patients initially treated with HFNO remained with a PaO_2_/FiO_2_ ratio ≤ 300 after intubation [17]. All this evidence led to the addition of HFNO in a recently published more global definition of ARDS [18]. The inclusion of HFNO patients would allow us to identify patients with ARDS in earlier stages of lung injury and, consequently, to test treatments prior to intubation and mechanical ventilation (MV). It will also broaden the applicability of the ARDS definition by focusing on all patients with clinically significant lung injury requiring high levels of oxygen support and including those patients treated in resource-limited settings.

Patients treated with HFNO may present better outcomes than MV ARDS patients [17, 19]. These differences in outcomes may be, at least partially, explained by the presence of different subphenotypes. However, the characterization of subphenotypes in patients with ARDS treated with HFNO has not been explored. We hypothesized that, as happen in MV ARDS patients, ARDS patients treated with HFNO might present different subphenotypes with different outcomes. Therefore, we aimed to identify inflammatory subphenotypes in a prospective cohort of ARDS patients treated with HFNO.

## Methods

We conducted a post hoc analysis of a multicenter prospective observational study comparing plasma biomarkers in mechanically ventilated and non-intubated ARDS patients [15]. Clinical data from the original cohort have been published elsewhere [15]. Briefly, patients were enrolled between 2014 and 2016 across 3 general ICU in tertiary hospitals in Spain.

In the present study inclusion criteria were non-intubated patients, treated with HFNO for hypoxemia defined by PaO_2_/FiO_2_ ≤ 300 or pulse oximetry (SpO_2_)/FiO_2_ ≤ 315, with bilateral radiographic opacities not fully explained by cardiac failure. FiO_2_ was set to ensure a SpO_2_ > 92%. The flow was adjusted according to patient tolerance. Need for MV was left at the discretion of the attending physician. Ethics Committee of each hospital approved the study. Written consent from patients or their relatives was obtained.

Baseline-recorded data included demographic characteristics, comorbidities, and etiology of ARDS. General respiratory and hemodynamic variables were collected at inclusion. Severity was assessed with the Acute Physiology and Chronic Health Evaluation (APACHE) II score within 24 h of ICU admission.

Sequential Organ Failure Assessment (SOFA) at ARDS onset while treated with HFNO was calculated. Acute renal failure was defined as a serum level of creatinine of 1.2 mg/dL or higher, and shock was defined by use of vasopressors. Follow-up for survival was performed during the 60 days after inclusion.

Blood samples were collected within 24 h of ARDS onset while being treated with HFNO. Plasma biomarkers of lung epithelial (receptor for advanced glycation end products [RAGE] and surfactant protein D [SP-D]) and endothelial (angiopoietin-2 [Ang-2]) injury as well as inflammation markers (interleukin [IL]-6, IL-8, and IL-33 and soluble suppression of tumorigenicity-2 [sST2]) levels were measured using enzyme-linked immunosorbent assay kits.

A detailed description of the statistical analysis is provided in the additional material (Additional file 1). Briefly, missing data were imputed using multiple imputation. sST2, IL-6, IL-8, angiopoietin-2, and s-RAGE were log10. Cluster analysis by K-means was used on the following variables: IL-33, sST2 (log), IL-6 (log), IL-8 (log), SP-D, angiopoietin-2 (log), RAGE (log), and bicarbonate level. A heatmap was used to represent the contribution of each biomarker to cluster identification. Values range from −1 to 1. A value close to zero has little or no impact on cluster attribution, while being close to 1 or −1 means a high impact on cluster attribution. The sign of the value indicates how biomarker’s variation influences cluster attribution (positive meaning that an increase in the value is correlated with cluster attribution). NbClust package was used to determine the optimal number of cluster [20]. Stability index was calculated by bootstrapping. After cluster attribution, patients were compared for initial characteristics and outcomes. Continuous variables were expressed as median [inter-quartile] and compared with Wilcoxon test. Categorical variables were reported as frequency (percentage) and compared with Chi-square.

To reduce the number of dimensions of our model, logistic regression was performed on all possible variable combinations to predict cluster belonging. We chose the model with the best area under the curve (AUC) of the receiving operative characteristics (ROC) and the lowest number of variables. A score to predict cluster belonging was designed by multiplying the variable's value by its corresponding odds ratio in the chosen model. The score threshold was then determined to achieve the optimal sensitivity and specificity [21].

Statistical analyses were conducted using R software (version 4.2.2). Results were considered statistically significant for a *p* value < 0.05*.*

## Results

### Study population

During a 3-year period, 170 ARDS patients were enrolled in the original cohort [15]. Among those patients, 127 were under mechanical ventilation at inclusion and, therefore, they were not included in the present study. Forty-three patients were initially treated with HFNO, of whom 41 had measurement of biomarkers at inclusion. The baseline characteristics of the non-intubated ARDS population treated with HFNO included are presented in Table 1.

**Table 1:** Baseline characteristics

	Overall (*n* = 41)	Hypoinflammatory subphenotype (*n* = 24)	Hyperinflammatory Subphenotype (*n* = 17)	*p* value
Sex (female), *n* (%)	10 (24.4)	4 (16.7)	6 (35.3)	0.318
Age (year), median [IQR]	60 [50, 68]	61 [50, 67]	60 [51, 72]	0.761
Comorbidities, *n* (%)				
Arterial hypertension	18 (43.9)	14 (58.3)	4 (23.5)	0.058
Diabetes	12 (29.3)	8 (33.3)	4 (23.5)	0.740
Cardiovascular diseases	6 (14.6)	4 (16.7)	2 (11.8)	1.000
COPD	4 (9.8)	2 (8.3)	2 (11.8)	1.000
ARDS etiology, *n* (%)				0.148
Pneumonia	31 (75.6)	19 (79.2)	12 (70.6)	
Other	5 (12.2)	4 (16.7)	1 (5.9)	
Pancreatitis	2 (4.9)	1 (4.2)	1 (5.9)	
Extrapulmonary sepsis	3 (7.3)	0 (0.0)	3 (17.6)	
APACHE_II, median [IQR])	18 [14, 22]	15 [13, 21]	20 [17, 22]	0.058
At the time of ARDS diagnosis				
SOFA, median [IQR]	6 [4, 8]	6 [4, 8]	6 [5, 8]	0.758
PaO_2_/FiO_2_, median [IQR]	96 [79, 116]	86 [70, 114]	107 [88, 125]	0.179
FiO_2_, median [IQR]	0.85 [0.70, 1]	0.80 [0.70, 1]	0.85 [0.60, 1]	0.742
Flow (L min^−1^), median [IQR]	60 [53, 60]	60 [53, 60]	60 [55, 60]	0.844
Respiratory rate, median [IQR]	26 [21, 32]	24 [21, 30]	30 [25, 34]	0.107
pH, median [IQR]	7.43 [7.40, 7.47]	7.44 [7.41, 7.47]	7.43 [7.39, 7.44]	0.087
PaCO_2_ (mmHg), median [IQR]	36 [32, 43]	36 [32, 40]	37 [31, 44]	0.750
Leukocytes (**×**10^6^ L^−1^), median [IQR]	11.1 [7.2, 14.3]	11.8 [7.8, 14.5]	8.2 [4.0, 13.5]	0.361

*COPD* Chronic obstructive pulmonary disease, *APACHE* Acute Physiology and Chronic Health Evaluation, *ARDS* Acute respiratory distress syndrome, *SOFA* Sequential Organ Failure Assessment. Data are expressed as median [IQR] or frequency (percentage)

### Identification of subphenotypes

**Fig. 1:**
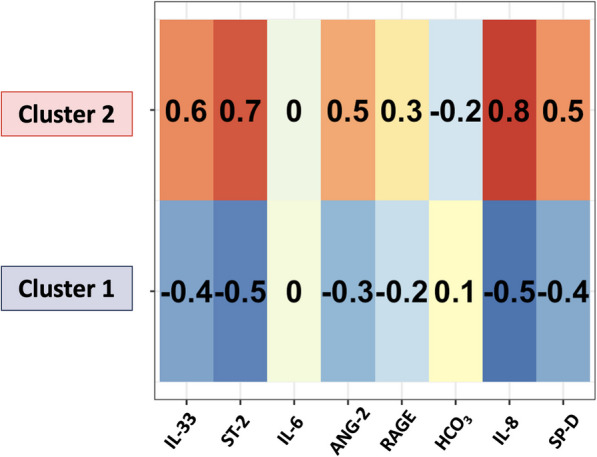
Heat map representing the strength of each variable to influence cluster belonging. Values range from −1 to 1. A value close to zero has little or no impact on cluster attribution, whereas a value close to 1 or −1 indicates a high impact on cluster attribution. The sign of the value indicates how the biomarker variation influences cluster attribution (when positive, an increase in the value is correlated with cluster attribution). IL: interleukin; sST2: soluble suppression of tumorigenicity-2; SP-D: surfactant protein D; RAGE: receptor for advanced glycation end products; ANG-2: angiopoietin; HCO3: bicarbonate

The correlation matrix did not identify any couple of variables with a correlation coefficient R ≥ 0,50. The optimal number of clusters was defined as two. Using unsupervised clustering analysis and considering the plasma concentration of the previously mentioned biomarkers, we identified 2 clusters of patients within the ARDS patients treated with HFNO (see Additional file 2). The two clusters were well separated with no overlap (see Additional file 3). The contribution of each biomarker to cluster identification was depicted using a heatmap (Fig. 1). IL8 and sST2 are the two biomarkers that differ more between clusters. The stability of our model was evaluated by calculating the stability index by bootstrapping 200 times the samples. The stability index achieved a value of 0.63.

### Clinical and biological characteristics and outcomes of the two subphenotypes

**Fig. 2:**
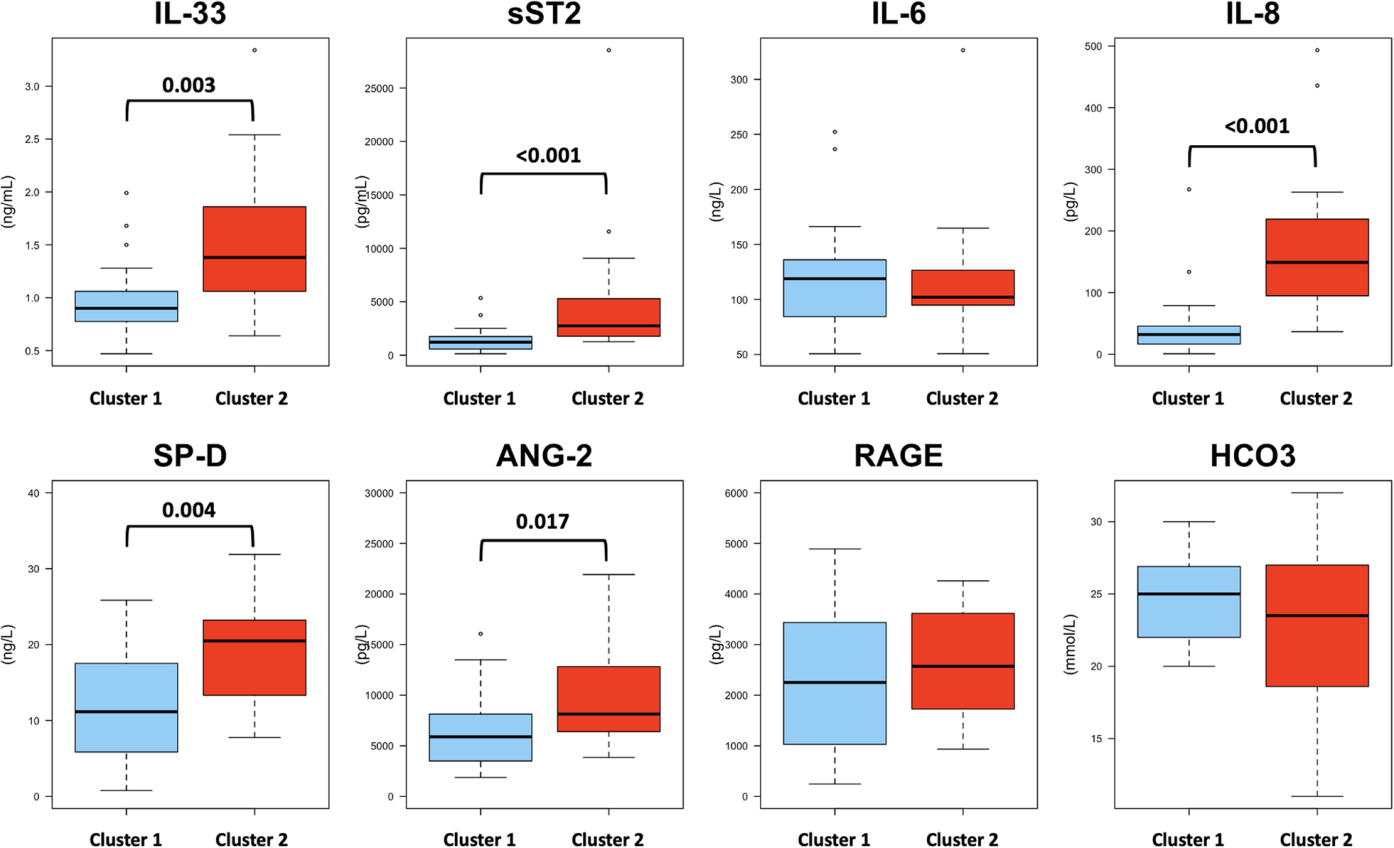
Comparison of biomarkers levels between clusters. IL: interleukin; sST2: soluble suppression of tumorigenicity-2; SP-D: surfactant protein D; RAGE: receptor for advanced glycation end products

We defined cluster 2 as the hyperinflammatory subphenotype as they had significantly increased levels of IL-33, sST2, IL-8, SP-D, and ANG-2 compared to cluster 1 (Fig. 2 and see Additional file 4).

No differences were found in IL-6, RAGE, and bicarbonate (HCO_3_). Cluster 1 was consequently defined as the hypoinflammatory subphenotype. No differences in baseline clinical characteristics were observed between both subphenotypes (Table 1).

The need for MV in the hyperinflammatory subphenotype was 64.7 versus 33.3% in the hypoinflammatory (*p* = 0.096). Patients belonging to the hyperinflammatory subphenotype were more frequently intubated at day 7 compared to the hypoinflammatory subphenotype (7 (30.4%) versus 9 (52.9%), *p* = 0.049). The ICU length of stay was longer in the hyperinflammatory subphenotype (22.0 days [18.0, 30.0] versus 39.5 [25.5, 60.0], *p* = 0.034). The hyperinflammatory patients had higher 60-day mortality compared to hypoinflammatory patients (47.1% vs. 9.7%; *p* = 0.020) (Table 2).

**Table 2:** Clinical outcomes during ICU course

	Overall (*n* = 41)	Hypoinflammatory subphenotype (*n* = 24)	Hyperinflammatory subphenotype (*n* = 17)	*p* value
Shock, *n* (%)	22 (53.7)	11 (45.8)	11 (64.7)	0.381
Renal failure, *n* (%)	21 (51.2)	10 (41.7)	11 (64.7)	0.256
Need for MV during ICU course, *n* (%)	19 (46.3)	8 (33.3)	11 (64.7)	0.096
Need for MV at day 3, *n* (%)	14 (35.0)	6 (26.1)	8 (47.1)	0.299
Need for MV at day 7, n (%)	16 (40.0)	7 (30.4)	9 (52.9)	0.049
Days of MV, median (IQR) (all patients)	8.5 [6.0, 22.0]	9.0 [5.0, 23.0]	8.0 [7.0, 20.0]	0.916
Days of MV, median (IQR) (survivors)	8.5 [6.5, 20.8]	9.0 [5.0, 23.0]	8.0 [7.5, 14.0]	1.000
ICU length of stay, median (IQR)	28.0 [18.5, 56.5]	22.0 [18.0, 30.0]	39.5 [25.5, 60.0]	0.034
60-day mortality, *n* (%) *n* = 39	10 (25.6)	2 (9.1)	8 (47.1)	0.020

*MV* mechanical ventilation, *ICU* intensive care unit. Data are expressed as median [IQR] or frequency (percentage)

### Subphenotype prediction with reduced model

**Fig. 3:**
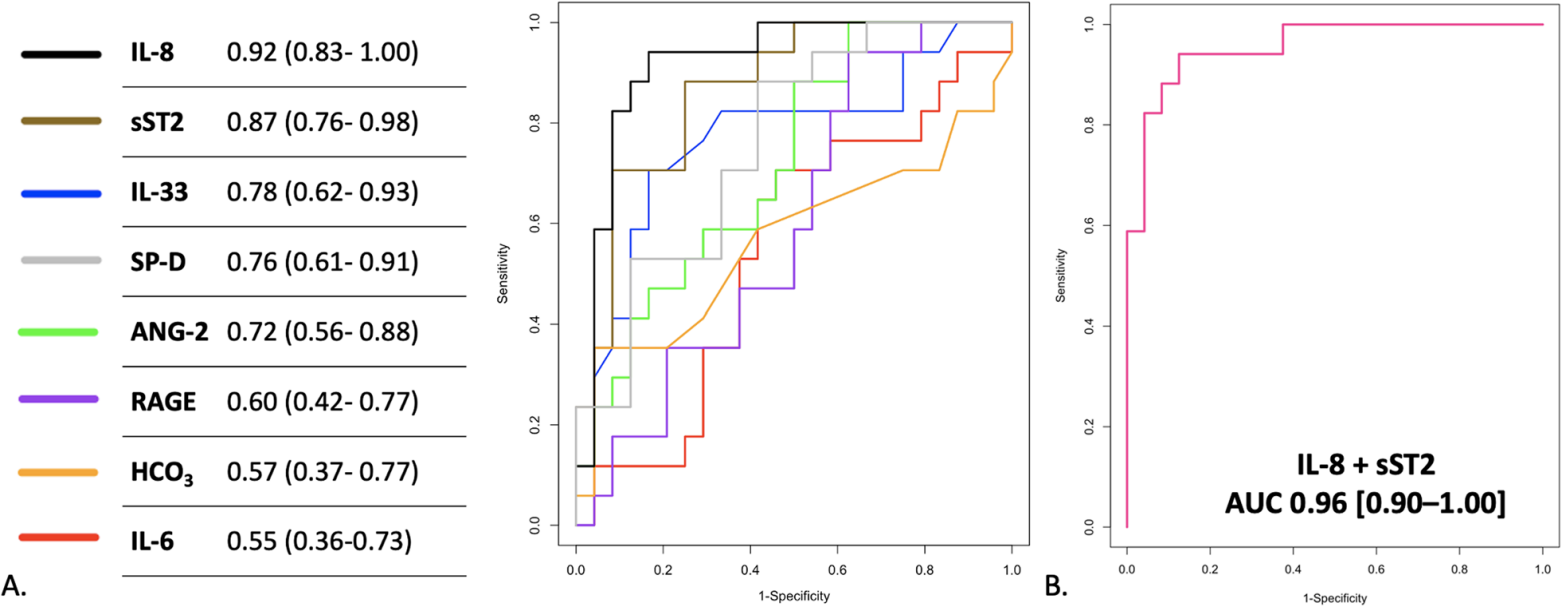
Diagnostic accuracy for subphenotype identification of different biomarkers. **A** ROC curves describing the ability of each variable to predict cluster appurtenance. **B** ROC curve of the IL-8 + sST2 model

The ROC curves illustrating the capacity of each biomarker to predict cluster appurtenance are presented in Fig. 3A. IL-8 alone had an area under the ROC curve of 0.92 (0.83–1.00) for cluster prediction. Using a dimension reduction model by logistic regression analysis, IL-8 and sST2 were able to accurately predict the two subphenotypes (area under the ROC curve of 0.96 [95%-CI: 0.90–1.00]) (Fig. 3B).

A simple score based on the plasma concentration of IL-8 and sST2, named HAIS score (High-flow ARDS Inflammatory Subphenotype), was able to determine the subphenotype of each patient (HAIS score = 1.48* log10([IL-8]) + 1.52* log10([ST-2])). A HAIS score ≥ 7.45 indicates that the patient belongs to subphenotype 2 with a sensitivity of 0.87 and a specificity of 0.94. It has a positive and negative predictive value of 0.95 and 0.84, respectively.

## Discussion

This is the first study that analyzed the presence of subphenotypes in non-intubated ARDS patients treated with HFNO. Based on inflammatory biomarkers, we identified two different subphenotypes that, despite presenting similar clinical characteristics at baseline, had divergent outcomes. Indeed, patients of hyperinflammatory subphenotype had higher mortality and longer ICU length of stay. Moreover, a score based on 2 inflammatory biomarkers (sT2 and IL-8) can accurately identify the subphenotype of HFNO patients who met ARDS criteria.

ARDS is characterized by a pulmonary and systemic inflammatory response which injures lung epithelium and endothelium resulting in protein-rich pulmonary edema [22]. Traditionally, the use of biomarkers has provided valuable insights into ARDS pathophysiology and has been used for risk stratification. Higher plasma concentrations of RAGE and SP-D, which are markers of epithelial injury, have been observed in ARDS patients [23, 24]. Similarly, elevated plasma Ang-2 predicts ARDS development in MV patients [25]. More recent studies have also shown the importance of the IL33/ST2 axis in the generation and modulation of lung injury [26, 27]. Finally, it has also been shown how these and other biomarkers, such as IL6 or IL8, are associated with outcomes, in both MV [28] and HFNO [15] ARDS patients. Interestingly, HFNO and MV ARDS patients have similar plasma levels of inflammatory biomarkers, suggesting that both have the same underlying pathophysiological alterations [15].

Subphenotypes in ARDS patients were first described by Calfee et al. [2] using latent class analysis of two retrospective cohorts of ARDS patients previously included in two randomized controlled trials. Subsequently, the same subphenotypes were observed in other secondary analyses of randomized controlled trials [3–5] and also in unselected populations of ARDS patients [6, 7, 9]. All studies agreed on the description of two subphenotypes that have different outcomes. As differences between subphenotypes were mainly driven by plasma levels of different biomarkers, they were named as the hyperinflammatory subphenotype, which had a higher mortality, and the hypoinflammatory subphenotype, which had a lower mortality rate. The subphenotype was typically evaluated on day one of the ARDS diagnosis. However, it has also been shown that ARDS subphenotypes are stable over the first three days of enrollment in the trial [8]. Finally, subphenotypes responded differently to various therapeutic interventions. In this sense, patients with the hyperinflammatory subphenotype have a lower mortality rate when treated with higher PEEP [2], a conservative fluid strategy [3], or simvastatin [4], suggesting that phenotyping ARDS patients may be a useful enrichment strategy to increase the likelihood of positive results in clinical trials with MV ARDS patients.

In contrast, most studies on HFNO have focused on identifying predictors of intubation based on patients’ clinical characteristics. Indeed, no studies have attempted to identify HFNO patients with a higher risk of death. To our knowledge, it is the first time that subphenotypes are identified across non-intubated ARDS patients. Indeed, another prospective observational study (NCT04009330) is currently recruiting patients to study ARDS subphenotypes in classical MV ARDS and in non-intubated ARDS. Importantly, this is the first time that we have been able to predict mortality in patients with HFNO. It should also be noted that our results are consistent with the subphenotypes observed in MV ARDS and their association with mortality. The identification of subphenotypes may therefore enhance enrichment strategies in clinical trials involving HFNO patients aiming to reduce the heterogeneity of treatment effect and to increase the likelihood of benefit of any given treatment.

The present study has some limitations. First, it is a secondary analysis that included a relatively small sample size. However, for k-means clustering analysis, the power to detect clustering primarily depends on cluster separation rather than on sample size [29]. Indeed, 20 observations per subgroup resulted in sufficient power to detect the presence of subgroups with k-means, also providing near-perfect accuracy for detecting the true number of clusters, and very high classification accuracy of individual observation’s group membership. Moreover, it is a unique cohort of HFNO patients in whom a panel of biomarker concentration has been determined. Second, our results need to be prospectively validated in an external cohort and the stability over time of these subphenotypes in HFNO patients should also be assessed. However, the results presented are consistent with previously reported data on MV ARDS patients [2–7, 9]. Third, the number of biomarkers compared to the number of patients exposed to the risk of overfitting. Nevertheless, this risk is compensated by the fact that each biomarker we used has been highly validated in MV ARDS studies, giving information on prognosis, or allowing the identification of subphenotypes. Fourth, the post hoc design of our study does not allow to study the correlation between biological subphenotypes and other clinical features such as radiological phenotype (diffuse or focal) or the delay between the first respiratory symptoms and non-intubated ARDS diagnosis. Likewise, as SpO_2_ was not recorded, we could not calculate the ROX index. However, PaO_2_/FiO_2_ ratio and RR were similar between groups. Therefore, it is unlikely that significant differences existed in the ROX index between groups.

In conclusion, using cluster analysis on inflammatory biomarkers we identified two subphenotypes in non-intubated ARDS patients under HFNO. These subphenotypes were indistinguishable according to the baseline clinical characteristics. However, they had radically different outcomes. A score based on sST2 and IL-8 serum concentrations can identify these subphenotypes with excellent accuracy and might be used for enrichment strategies in future clinical trials involving HFNO patients.

### Supplementary Information

**Additional file 1.** Detailed statistical analysis.

**Additional file 2.** Variables included in cluster analysis.

**Additional file 3.** Cluster plot.

**Additional file 4.** Plasma concentration of the different biomarkers.

## Data Availability

The datasets used and/or analyzed during the current study are available from the corresponding author on reasonable request.

## Abbreviations

Ang-2: Angiopoietin-2
APACHE: Acute Physiology and Chronic Health Evaluation
ARDS: Acute respiratory distress syndrome
AUROC: Area under the curve of the receiving operative characteristics
HAIS score: High-flow ARDS Inflammatory Subphenotype score
HCO_3_: Bicarbonate
HFNO: High-flow nasal oxygen
ICU: Intensive care unit
IL-6: Interleukin-6
IL-8: Interleukin-8
IL-33: Interleukin-33
MV: Mechanical ventilation
PaO_2_/FiO_2_: Partial pressure of oxygen in arterial blood to the fraction of inspiratory oxygen concentration ratio
PEEP: Positive end-expiratory pressure
RAGE: Receptor for advanced glycation end products
SOFA: Sequential Organ Failure Assessment
SP-D: Surfactant protein D
SpO2: Peripheral oxygen saturation
sST2: Soluble suppression of tumorigenicity-2
