# Supplementary material for: Subphenotypes in patients with acute respiratory distress syndrome treated with high-flow oxygen
Source: Crit Care. 2023 Nov 1;27:419. doi: 10.1186/s13054-023-04687-0 (PMC10619276; doi:10.1186/s13054-023-04687-0)
Supplement: Supplementary file 1 — Additional file 1. Detailed statistical analysis. [file 13054_2023_4687_MOESM1_ESM.docx]

# Additional file 1.

## Detailed statistical analysis

Prior to beginning the analysis, clinical variables were examined for missing data, distribution, and correlation. Few data were missing (2.5%) with no identifiable pattern (i.e. missing at random). Missing data were imputed using multiple imputation by predictive mean matching^1^. Variables with high variance (sST2, IL-6, IL-8, angiopoietin-2 and s-RAGE) were log10 transformed. A correlation matrix was drawn to suppress variables that were too correlated (correlation coefficient > 0.5).

Clustering analysis was conducted using the K-means method on the following variables: IL-33, sST2 (log), IL-6 (log), IL-8 (log), SP-D, angiopoietin-2 (log), and RAGE (log). Bicarbonate level was also included, as it was previously demonstrated to be a strong indicator of cluster belonging^2–4^. K-means is an unsupervised machine learning algorithm used to cluster data points into clusters based on their similarity. It calculates the distance between each data point and the centroids and assigns each data point to the closest centroid. NbClust package was used to define the number of clusters^5^. This method has been done in similar study by Bos et al^6^. The stability of our results was computed by bootstrapping K-means algorithm. Once the optimal number of clusters was determined, patients were compared for initial characteristics and outcomes based on their cluster attribution. Continuous variables were expressed as median [inter-quartile] and were compared with Wilcoxon-test. Categorial variables were reported as frequency (percentage) and were compared with Chi-square.

Dimension reduction of our model was conducted by using logistic regression. After ensuring that conditions to use multiple logistic regression were filled, all possible combinations of variables were defined. A logistic regression on these combinations was performed to predict cluster belonging. We calculated for each regression the area under the received operating characteristic (ROC) curve associated with the model to predict who belongs to each cluster. ROC curves of each variable taken individually, and ROC curves of the best models composed of the fewest number of variables were drawn. The model with the best balance between the lowest number of variables and the best AUC was chosen. Finally, we extracted the odds ratio associated with each variable and calculated a score by multiplying the variable's value by its corresponding odds ratio in the model. The score threshold was then determined to achieve the optimal balance between sensitivity and specificity for classifying data into cluster 1 or cluster 2.

Statistical analyses were conducted using R software (version 4.2.2). Results were considered statistically significant for a p-value < 0.05*.* **REFERENCES**

1. Package “mice.” Accessed August 2, 2023. https://cran.r-project.org/web/packages/mice/mice.pdf

2. Calfee CS, Delucchi K, Parsons PE, Thompson BT, Ware LB, Matthay MA. Subphenotypes in acute respiratory distress syndrome: latent class analysis of data from two randomised controlled trials. *The Lancet Respiratory Medicine*. 2014;2(8):611-620. doi:10.1016/S2213-2600(14)70097-9

3. Calfee CS, Delucchi KL, Sinha P, et al. Acute respiratory distress syndrome subphenotypes and differential response to simvastatin: secondary analysis of a randomised controlled trial. *The Lancet Respiratory Medicine*. 2018;6(9):691-698. doi:10.1016/S2213-2600(18)30177-2

4. Famous KR, Delucchi K, Ware LB, et al. Acute Respiratory Distress Syndrome Subphenotypes Respond Differently to Randomized Fluid Management Strategy. *Am J Respir Crit Care Med*. 2017;195(3):331-338. doi:10.1164/rccm.201603-0645OC

5. NbClust.pdf. Accessed August 2, 2023. https://cran.r-project.org/web/packages/NbClust/NbClust.pdf

6. Bos LD, Schouten LR, van Vught LA, et al. Identification and validation of distinct biological phenotypes in patients with acute respiratory distress syndrome by cluster analysis. *Thorax*. 2017;72(10):876-883. doi:10.1136/thoraxjnl-2016-209719
