# Supplementary material for: Subphenotypes in patients with acute respiratory distress syndrome treated with high-flow oxygen
Source: Crit Care. 2023 Nov 1;27:419. doi: 10.1186/s13054-023-04687-0 (PMC10619276; doi:10.1186/s13054-023-04687-0)
Supplement: Supplementary file 2 — Additional file 2. Variables included in cluster analysis. [file 13054_2023_4687_MOESM2_ESM.docx]

**Additional file 2. Variables included in Cluster analysis**

|  | Overall  (n=41) | | Hypo-inflammatory subphenotype  (n= 24) | | Hyper-inflammatory subphenotype  (n=17) | p-value |
| --- | --- | --- | --- | --- | --- | --- |
| IL-33 (ng/mL), median [IQR]) | | 1.05 [0.81, 1.43] | | 0.90 [0.79, 1.06] | 1.38 [1.06, 1.86] | 0.003 |
| Log_sST2 (ρg/mL), median (IQR) | | 3.22 [2.99, 3.44] | | 3.09 [2.76, 3.22] | 3.44 [3.25, 3.72] | <0.001 |
| Log_IL-6 (ng/L), median (IQR) | | 2.04 [1.97, 2.13] | | 2.07 [1.93, 2.13] | 2.01 [1.98, 2.10] | 0.628 |
| Log_IL-8 (ρg/mL), median (IQR) | | 1.69 [1.45, 2.12] | | 1.50 [1.23, 1.64] | 2.17 [1.98, 2.34] | <0.001 |
| SP-D (ng/mL), median (IQR) | | 13.70 [8.47, 21.29] | | 11.15 [6.48, 16.97] | 20.49 [13.31, 23.22] | 0.004 |
| Log_RAGE (ρg/mL), median (IQR) | | 3.38 [3.11, 3.55] | | 3.35 [3.01, 3.53] | 3.41 [3.24, 3.56] | 0.279 |
| Log_ANG-2 (ρg/mL), median (IQR) | | 3.83 [3.62, 4.04] | | 3.77 [3.54, 3.89] | 3.91 [3.81, 4.11] | 0.017 |
| HCO_3_ (mmol/L), median (IQR) | | 24.5 [20.0, 26.7] | | 25.0 [22.0, 26.5] | 23.0 [18.1, 26.5] | 0.338 |
| IL: interleukin; sST2: soluble suppression of tumorigenicity-2; SP-D: surfactant protein D; RAGE: receptor for advanced glycation end products; ANG-2: Angiopoietin; HCO_3_: bicarbonate. Data are expressed as median [IQR] | | | | | | |
