# Supplementary figures and images for: Subphenotypes in patients with acute respiratory distress syndrome treated with high-flow oxygen
Source: Crit Care. 2023 Nov 1;27:419. doi: 10.1186/s13054-023-04687-0 (PMC10619276; doi:10.1186/s13054-023-04687-0)

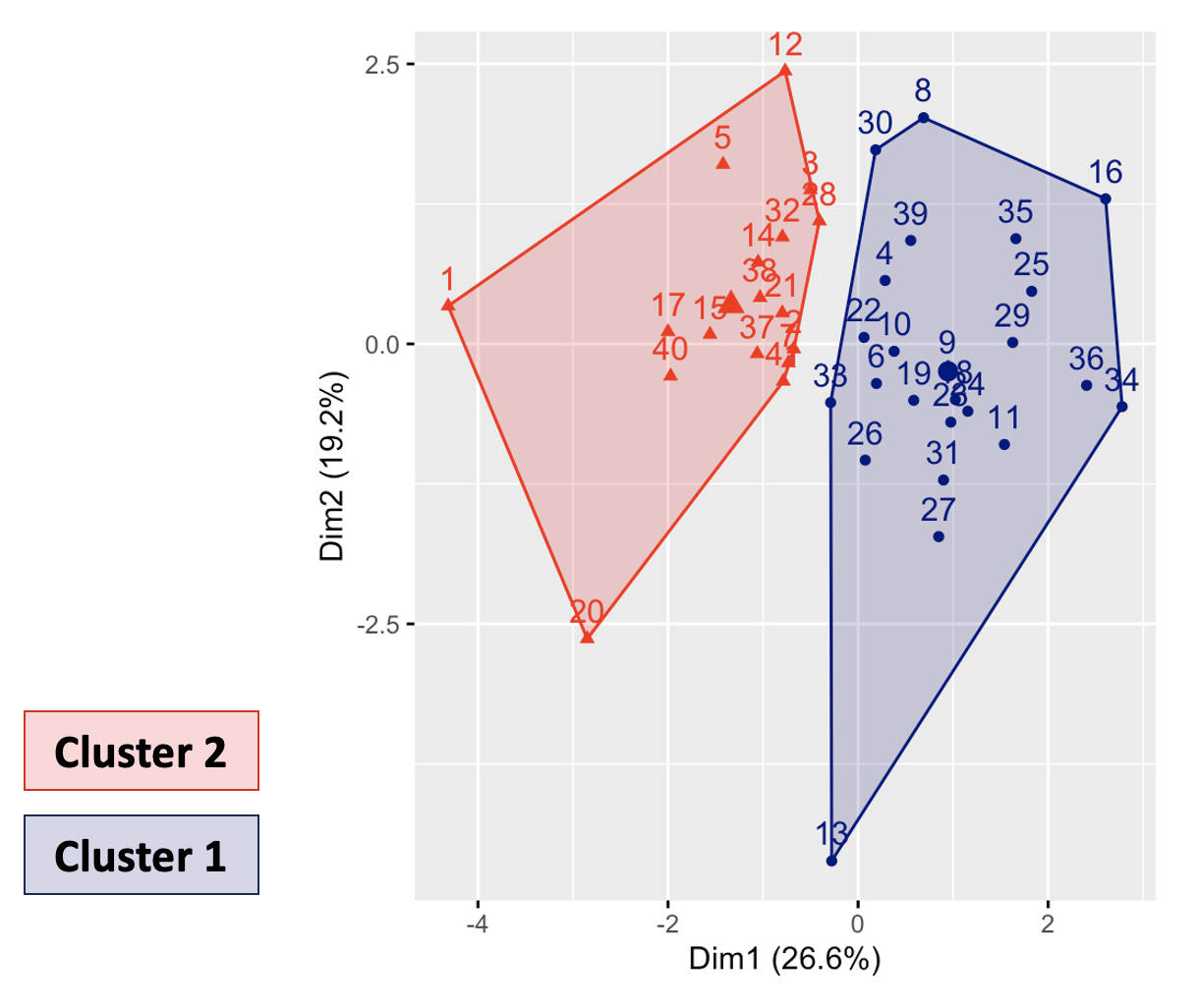

Supplement: Supplementary file 3 — Additional file 3. Cluster plot. [file 13054_2023_4687_MOESM3_ESM.tif]
