# Supplementary material for: Subphenotypes in patients with acute respiratory distress syndrome treated with high-flow oxygen
Source: Crit Care. 2023 Nov 1;27:419. doi: 10.1186/s13054-023-04687-0 (PMC10619276; doi:10.1186/s13054-023-04687-0)
Supplement: Supplementary file 4 — Additional file 4. Plasma concentration of the different biomarkers. [file 13054_2023_4687_MOESM4_ESM.docx]

**Additional file 4. Plasma concentration of the different biomarkers**

|  | Overall  (n=41) | Hypo-inflammatory subphenotype  (n= 24) | Hyper-inflammatory subphenotype  (n=17) | p-value |
| --- | --- | --- | --- | --- |
| IL-33 (ng/mL), median [IQR]) | 1.05 [0.81, 1.43] | 0.90 [0.79, 1.06] | 1.38 [1.06, 1.86] | 0.003 |
| sST2 (ρg/mL), median (IQR) | 1652 [982, 2748] | 1220 [579, 1664] | 2756 [1782, 5195] | <0.001 |
| IL-6 (ng/L), median (IQR) | 109 [94, 135] | 119 [85, 136] | 102 [95, 127] | 0.628 |
| IL-8 (ρg/mL), median (IQR) | 49.5 [28.0, 132.0] | 31.8 [17.0, 43.7] | 149.0 [94.9, 219.3] | <0.001 |
| SP-D (ng/mL), median (IQR) | 13.70 [8.47, 21.29] | 11.15 [6.48, 16.97] | 20.49 [13.31, 23.22] | 0.004 |
| RAGE (ρg/mL), median (IQR) | 2394 [1289, 3579] | 2251 [1031, 3394] | 2571 [1740, 3615] | 0.279 |
| ANG-2 (ρg/mL), median (IQR) | 6722 [4193, 10970] | 5890[3501, 7848] | 8135 [6398, 12825] | 0.017 |
| IL: interleukin; sST2: soluble suppression of tumorigenicity-2; SP-D: surfactant protein D; RAGE: receptor for advanced glycation end products; ANG-2: Angiopoietin; HCO_3_: bicarbonate. Data are expressed as median [IQR] | | | | |
